# Fear of COVID-19 and Depression: A Comparative Study Among the General Population and Healthcare Professionals During COVID-19 Pandemic Crisis in Bangladesh

**DOI:** 10.1007/s11469-020-00477-9

**Published:** 2021-02-19

**Authors:** Najmuj Sakib, Tahmina Akter, Fatematuz Zohra, A. K. M. Israfil Bhuiyan, Mohammed A. Mamun, Mark D. Griffiths

**Affiliations:** 1 Centre for Health Innovation, Networking, Training, Action and Research - Bangladesh (CHINTA Research Bangladesh), Dhaka, Bangladesh; 2 Department of Microbiology, Jashore University of Science and Technology, Jashore, Bangladesh; 3 Department of Epidemiology, Bangladesh University of Health Sciences, Dhaka, Bangladesh; 4 Department of One Health, Chittagong Veterinary and Animal Sciences University, Chittagong, Bangladesh; 5 Department of Public Health and Informatics, Jahangirnagar University, Savar, Dhaka, Bangladesh; 6 Psychology Department, Nottingham Trent University, Shakespeare Street, Nottingham, UK

**Keywords:** Depression, Fear, COVID-19, Psychological impacts, Healthcare professionals, Bangladesh

## Abstract

The COVID-19 pandemic affects individuals’ mental health that can result in fear of getting COVID-19 infection and depression. As there is no prior study available, we evaluated these mental health outcomes and associated factors among the general population and healthcare professionals (HCPs) in Bangladesh. This nationwide cross-sectional study comprised 3388 individuals including 834 HCPs. The measures included socio-demographics, healthcare, and patient-care related information, the Bangla Patient Health Questionnaire, and the Bangla Fear of COVID-19 Scale. Multiple linear regression analyses were performed to identify risk factors. Just over one-quarter of the participants were depressed, and was significantly associated with COVID-19 fear. Regression analyses showed that, both in general population and HCPs, depression and fear of COVID-19 were strongly predicted by being female; however, depression was inversely associated with being married. Particularly, among the HCPs, being restless while examining a patient with flu-like symptoms and while examining a patient returning from abroad was found to be significant predictor for both depression and fear of COVID-19. HCPs who were using single protective equipment for a week had greater depression and those who felt insecure due to the pandemic had a high level of COVID-19 fear. The findings identified major psychological impacts among the participants, suggesting the urgent need to promote mental wellbeing in both general population and medical professionals.

Many countries including Bangladesh are undergoing an unprecedented health emergency due to the spread of the novel coronavirus 2019 (COVID-19). The virus appears to have originated in a seafood market in China in December 2019 (Yu et al. 2020). The virus has spread to almost all countries and territories and was declared a pandemic by the World Health Organization in March 2020. At the time of writing there have been over 57.33 million confirmed cases around the world and over 1.63 million deaths (World Health Organization, 2020a). On 18 March 2020, the first COVID-19 death was confirmed in Bangladesh (10 days after reporting the first case) (World Health Organization 2020b). It has now spread all over the country and the Bangladeshi government has declared the entire country at risk of COVID-19 (BBC News 2020). In March 2020, the percentage of new infections in Bangladesh was low, but since April 2020, it has continuously risen with over 25,000 confirmed cases and over 370 deaths as of May 20 (Institute of Epidemiology, Disease Control and Research 2020). The first death case of a frontline healthcare professional (HCP) was on April 15, 2020, which highlighted the fragile condition of the healthcare system of Bangladesh (Islam & Rabbi 2020).

However, extreme fear when combined with social and economic consequences (e.g., loss of job, reduced earnings, relationship problems) has the capacity to facilitate irrational thinking within individuals that may lead to psychological distresses (Pakpour and Griffiths 2020; Xiang et al. 2020). In addition, the fear of death, loneliness, boredom, anxiety, depression, social isolation, contagion, and (in extreme cases) thoughts of suicide can result in long-term psychological effects among general population in the previous deadly virus outbreaks (Lee et al., 2018; Mak et al. 2009). Frontline healthcare workers are also vulnerable to temporary and long-term psychiatric symptoms due to the fear of being quarantined, fear of their family and friends being infected, caring for colleagues as a patient, feelings of stigmatization, and rejection by others in their neighborhood (Bai et al. 2004; Lee et al. 2007; Maunder et al. 2003). Many studies have reported that the general population as well as HCPs have been mentally affected by the COVID-19 pandemic (Pappa et al. 2020). In extreme cases, COVID-19-related suicides have been reported in Bangladesh (Bhuiyan et al. 2020; Mamun and Griffiths 2020) and other countries (Dsouza et al. 2020; Goyal et al. 2020; Griffiths and Mamun 2020; Mamun and Ullah 2020).

In Bangladesh, it has been reported that national disasters are not favorable to mental wellbeing and there is no mental health support programs in the country to combat such traumatic events (Hussain et al. 2011). For instance, a high rate of mental health suffering (i.e., a prevalence rate of 65% depression, much higher than rates reported elsewhere) has previously been reported in the aftermath of a natural disaster (Mamun et al. 2019), and appears to reflect the vulnerability of the Bangladeshi population to mental health suffering in a pandemic situation such as COVID-19. Like other countries, people in Bangladeshi rushed to shops to buy facemasks and sanitizers immediately after the first case of COVID-19 was confirmed in the country, and which led to elevated public panic (United News of Bangladesh 2020). Moreover, there appears to be an epidemic of fear toward affected or deceased individuals concerning the community transmission of COVID-19. For example, local residents from one area in Bangladesh protested against the burial of bodies who had died of COVID-19 in their community graveyard (Kamal 2020). The situation is of great concern in health sectors where many patients get admitted into private hospitals concealing their contact history, travel history, or flu-like symptoms, and these hospitals do not have the safety measures in place to deal with the COVID-19 patients (Anadolu Agency 2020). Consequently, many hospitals have to send their staff into quarantine and increasing numbers of hospital employees are infected with the virus which puts enormous strain on the Bangladeshi health system (Al Jazeera 2020). Such factors may have an influence on the mental stability of HCPs. Due to this critical situation, both the general population and frontline HCPs (i.e., those who are directly involved in the diagnosis, treatment, and care of patients with COVID-19) face daily psychological challenges and are at risk of experiencing mental health suffering (The Daily Star 2020).

Based on the current situation in Bangladesh, it is anticipated that individuals are already suffering from mental health issues, although there is no prior study concerning this in relation to COVID-19. Therefore, the present study assessed mental health burdens (i.e., depression and fear of COVID-19) among the Bangladeshi general population and HCPs. Given the exploratory nature of the study and the fact that no previous study has examined this specific cohort, there were no specific hypotheses.

## Methods

### Participants, Procedure, and Ethics

The present cross-sectional study was carried out between April 8 to April 25, 2020, among the general population (aged 18 years and older) and healthcare professionals (HCPs) of Bangladesh. An online-based survey was developed and participants were recruited via social media (e.g., *Facebook, WhatsApp, Twitter*). Approximately 4000 participants were approached and 3388 individuals took part in the survey (mean age 30.11 years; SD ± 6.44). Of these, 2554 were general population (29.62 years; SD ± 6.678) and 834 were HCPs (30.68 years; SD ± 5.61) who were directly involved in the treatment of COVID-19 patients. The respondents were only able to participate in the survey after they agreed to the online consent (that adhered to the guidelines of the Helsinki Declaration, 1975). All participants were assured of anonymity and confidentiality of their data, and were provided with information about the nature and purpose of the study, the procedure, and were informed about their right to retract their data at any time. In addition, formal ethical approval was obtained from the Institute of Allergy and Clinical Immunology of Bangladesh (Dhaka, Bangladesh) before the implementation of the study.

### Measures

#### Demographic Information

A contextual information sheet was used to obtain demographic and other information of the participants. The questions related to (i) age, (ii) gender, (iii) marital status (unmarried, married, or divorced), (iv) having children (yes/no), (v) having elderly people living at home who were at high-risk of COVID-19, and (vi) having chronic physical diseases (e.g., asthma, diabetics, heart diseases, chronic kidney disease, thyroid disorder).

#### Healthcare Facility and Patient-Care Related Information

HCPs were asked additional questions to examine whether healthcare-related factors had any influence on HCP’s depression and fear of COVID-19 (i.e., these questions were not asked to the general public). More specifically, they were asked: *“Do you purchase safety equipment for yourself?”*, *“Do you receive personal protective equipment (PPE) from your employer?”*, *“Do you need to reuse the purchased or the provided PPE?”*, and *“Are you satisfied with PPE quality?”.* All these questions were responded to utilizing a dichotomous (yes/no) response. One of three responses (i.e., “less than a week”, “a week”, or “more than a week”) was required for the question, “*How long do you use a single PPE?*” Additionally, patient-care related questions were asked concerning the attitude toward patients having flu-like symptoms or returning from abroad, the feeling of insecurity for oneself and family members, and the fear of spreading the disease to other family members (see Table 3 for list of exact statements asked). Finally, two (yes/no) questions were asked concerning (i) whether participants thought that having some kind of psychological interventions would improve their mental health, and (ii) whether participants felt prepared to tackle the COVID-19 disease crisis.

#### Bangla Patient Health Questionnaire

Participants’ health was assessed using the nine-item Bangla Patient Health Questionnaire (Bangla PHQ-9) (Chowdhury et al. 2004); original version, Kroenke, Spitzer, and Williams, 2001). The assessment tool is used widely in both non-psychiatric and clinical settings. Symptoms of depression such as depressed mood, sleeping problems, feeling of tiredness, changes of appetite, concentration problems, and suicidal thoughts are assessed based on the past 2 weeks. Items are responded to on a four-point Likert scale (0 = *not at all*, 1 = *several days,* 2 = *more than half of the days*, and 3 = *nearly every day*) and scores range from 0 to 27 (Kroenke et al. 2001)**.** Higher scores indicate higher levels of depression, whereas a total ≥ 10 is typically used to denote the presence of depression (Mamun et al. 2019). Cronbach’s alpha in the present study was 0.73.

#### Bangla Fear of COVID-19 Scale

The Bangla Fear of COVID-19 Scale (Sakib et al. 2020) assesses fear of COVID-19 and was adapted from the English version of the scale published in the original paper (Ahorsu et al. 2020). The screening tool consists of seven items (e.g., *“I cannot sleep because I am worried about getting coronavirus-19”*) with a five-item Likert-point response from 1 (*strongly disagree*) to 5 (*strongly agree*) and its score range is 7 to 35. The higher the score indicates the greater the fear of COVID-19 (Ahorsu et al. 2020). Cronbach’s alpha in the present study was 0.87.

### Data Analysis

The data were analyzed using Statistical Package for Social Science (SPSS) Version 25.0 for Windows (SPSS Inc., Chicago, IL, USA). Microsoft Excel 2016 was used for early data cleaning and grounding for SPSS format. The descriptive statistics (i.e., means, frequencies, percentages) were used for distribution of the variables across the general population and HCPs. Inferential statistics (e.g., chi-square tests) were performed to identify significant relationships between outcome variables (fear and depression) and the independent variables in groups (i.e., total population, general population, and HCPs). Multiple linear regressions were carried out to identify risk factors for depression and fear of COVID-19. Initially, all the variables were included in the regression model. Then, both forward selection and backward elimination methods were utilized to crosscheck and keep the predictors best fit to the models (e.g., gender, marital status, having children, having elderly people living at home who were at high-risk of COVID-19, the need to reuse purchased or provided PPE). The assumptions (i.e., linearity, normality, and homoscedasticity) with the data were checked for both the models before performing the regression analysis. Multicollinearity within the predictor variables was also checked with variance inflation factor (VIF) values. Variables with VIF values less than 5 were excluded from the final model. In this study, the regression models were interpreted with 95% confidence intervals and a *p*-value of <0.05 was considered statistically significant. The post-hoc statistical power observed for multiple-regression analysis was 1.0.

## Results

Of the total sample (*N* = 3388), just over half were females (*n* = 1754, 51.8%). The majority were married (60.8%), had no any children (57.8%), had elderly people who were at high-risk of COVID-19 living at home (66.1%), and had no chronic physical disease (57.5%). Three-quarters of the total sample were general population (75.4%) and the remainder were healthcare professionals (24.6%) (Table 1). Over half the HCPs were medical officers (53.2%), 10% were nurses, 6.8% were interns, 10% had affiliations with medical colleges (e.g., lecturer, assistant or associate professor, registrar), and the remainder held other positions not listed.

**Table 1:** Distribution of the variables across the total sample, general population sample, and healthcare professional sample

Variables	Total sample, *N* = 3388 (*n*; %)	General population (*n* = 2554, 75.4%)	Healthcare professionals (*n* = 834, 24.6%)
Gender			
Male	1634, 48.2%	1267, 49.6%	367, 44%
Female	1754, 51.8%	1287, 50.4%	467, 56%
Marital status			
Unmarried	1286, 38%	1028, 40.3%	258, 30.9%
Married	2059, 60.856%	1498, 58.7%	561, 67.3%
Divorced	43, 1.3%	28, 1.1%	15, 1.8%
Having children			
Yes	1429, 42.2%	1044, 40.9%	385, 46.2%
No	1959, 57.8%	1510, 59.1%	449, 53.8%
Having elderly people living at home who were at high-risk of COVID-19			
Yes	2240, 66.1%	1627, 63.7%	613, 73.5%
No	1148, 33.9%	927, 36.3%	221, 26.5%
Having chronic physical diseases			
Yes	1437, 42.4%	1110, 43.5%	327, 39.2
No	1951, 57.5%	1444, 46.5%	507, 60.8

Table 2 shows the prevalence rate of depression across the total sample, general population, and HCPs. Just over one-quarter of the participants were depressed (27.8%) in total and the depression ratio among the general population compared to HCPs was approximately 1:1 (see Table 2). Females in the total sample (34.1% vs 21.2%; χ^2^ = 70.238, *p* < .001) and in both general population (33.7% vs 22.3%; χ^2^ = 41.594, df = 1, *p* < .001) and HCPs (35.1% vs 17.4%; χ^2^ = 32.334; df = 1, *p* < .001) had a significantly higher prevalence of depression compared to males. With regard to marital status, individuals who were divorced had the highest prevalence of depression followed by those who were unmarried and married in the total sample (53.5%, 33.6%, 23.7%, respectively; χ^2^ = 52.387, *p* < .001), general population (50.0%, 34.6%, 23.1%, respectively; χ^2^ = 9.603, *p* = .008), and HCPs (60.0%, 29.5%, 25.5%, respectively; χ^2^ = 46.492, *p* < 0.001). Respondents with no children had a significantly higher prevalence of depression in the total sample and general population group (32.3% vs 21.8%; χ^2^ = 44.702, *p* < 0.001 and 33.2% vs 20.5%; χ^2^ = 49.711, *p* < .001, respectively) although, there was no significant difference among HCPs. Additionally, depression was significantly associated with having elderly people living at home who were at high-risk of COVID-19 and having chronic physical diseases within the total sample, general population, and HCPs (all *p* values <.001; Table 2).

**Table 2:** Distribution of the depression across the total sample, general population sample, and healthcare professional sample

Variables	Total (944; 27.8%)			General population (716, 28%)			Healthcare professionals (228, 27.3%)		
	Yes (%)	χ^2^ test value (df)	*p*-value	Yes (%)	χ^2^ test value (df)	*p*-value	Yes (%)	χ^2^ test value (df)	*p*-value
Gender									
Male	346, 21.2%	70.238 (1)	<.001*	282, 22.3%	41.594 (1)	<.001*	64, 17.4%	32.334 (1)	<.001*
Female	598, 34.1%			434, 33.7%			164, 35.1%		
Marital status									
Unmarried	432, 33.6%	52.387 (2)	<.001*	356, 34.6%	46.492 (2)	<.001*	76, 29.5%	9.603 (2)	.008
Married	489, 23.7%			346, 23.1%			143, 25.5%		
Divorced	23, 53.5%			14, 50.0%			9, 60.0%		
Having children									
Yes	312, 21.8%	44.702 (1)	<.001*	214, 20.5%	49.711 (1)	<.001*	98, 25.5%	1.277 (1)	.258
No	632, 32.3%			502, 33.2%			130, 29.0%		
Having elderly people living at home who were at high-risk of COVID-19									
Yes	686, 30.6%	25.090 (1)	<.001*	499, 30.7%	15.432 (1)	<.001*	187, 30.5%	11.685 (1)	<.001*
No	258, 22.5%			217, 23.4%			41, 18.6%		
Having chronic physical diseases									
Yes	507, 35.3%	68.332 (1)	<.001*	394, 35.5%	54.170	<.001*	113, 34.6%	14.110	<.001*
No	437, 22.4%			322, 22.3%			115, 22.7%		

*Statistically significant at < 0.004 level based on the Bonferroni correction to the *p*-value

Table 3 features the prevalence of depression among HCPs in relation to personal protective equipment they were using in their patient care. Approximately two-thirds of HCPs purchased their own safety equipment at least once (63.2%) and 49% had got PPE from their employer. Only 15.9% were satisfied with the quality of the PPE provided, and the prevalence of depression was higher among HCPs who were dissatisfied with their PPE (30.2% vs. 18.0%; χ^2^ = 7.964, *p* = .005). Two-thirds of HCPs were restless when examining patients with flu-like symptoms (65.3%) (33.0% depression vs 16.6% depression among those not restless; χ^2^ = 25.628, *p* < .001), 91.0% felt insecure for themselves and their family due to caring for COVID-19 patients (28.9% depression vs. 12.0% depression among those who did not feel insecure; χ^2^ = 9.760, *p* = .002), most of them (89.0%) believed that they were not ready for battling the COVID-19 pandemic (28.6% depression vs. 17.4% depression who felt they were ready; χ^2^ = 5.150, *p* = .023). Over nine-tenths of the HCPs (91.6%) believed that psychological interventions would improve their mental health and just under a half of HCPs kept a distance of 1 m from the patients who had recently returned from abroad (45%) (Table 3).

**Table 3:** Distribution of the healthcare facility and patient-care related information with depression among healthcare professionals (*n* = 834)

Variables	Total (*n*, %)	Depression (*n*, %)	χ^2^ test value	df	*p*-value
Purchased own PPE equipment					
Yes	527, 63.2%	150, 28.5%	0.912	1	.340
No	307, 36.8%	78, 25.4%			
Received PPE from employer					
Yes	409, 49%	111, 27.1%	0.016	1	.899
No	425, 51.0%	117, 27.5%			
Needed to reuse PPE					
Yes	360, 43.2%	82, 22.8%	7.346	1	.007
No	433, 51.9%	136, 31.4%			
Duration of using a single PPE					
Less than a week	316, 37.9%	80, 25.3%	3.540	2	.170
A week	136, 16.3%	46, 33.8%			
More than a week	239, 28.7%	70, 29.3%			
Satisfaction with PPE quality					
Yes	133, 15.9%	24, 18.0%	7.964	1	.005
No	603, 72.3%	182, 30.2%			
Restless when examining a patient with flu symptoms					
Yes	545, 65.3%	180, 33.0%	25.628	1	<.001*
No	289, 34.7%	48, 16.6%			
Insecure feeling for oneself and family members due to COVID-19					
Yes	759, 91%	219, 28.9%	9.760	1	.002
No	75, 9.0%	9, 12.0%			
Attitude toward a patient returning from abroad					
No difference	136, 16.3%	26, 19.1%	18.072	3	<.001*
Felt restless	273, 32.7%	98, 35.9%			
Maintain a distance of at least 1 m	375, 45.0%	88, 23.5%			
Disagree to examine the patient	50, 6.0%	16, 32.0%			
Maintained distance from family members due to fear of spreading the COVID-19 disease					
Put him/herself in complete isolation	186, 22.3%	48, 25.8%	13.869	3	.003
Use masks at home	33, 4.0%	0, 0.0%			
Maintain a distance of 1 m	200, 24.0%	60, 30.0%			
Do not follow any of the above	415, 49.8%	120, 28.9%			
Psychological sessions can improve mental health of HCPs					
Yes	764, 91.6%	207, 27.1%	0.273	1	.602
No	70, 8.4%	21, 30.0%			
Preparedness to tackle the COVID-19 disease crisis					
No	742, 89.0%	212, 28.6%	5.150	1	.023
Yes	92, 11.0%	16, 17.4%			

*Statistically significant at < 0.002 level based on the Bonferroni correction to the *p*-value

Multiple linear regression analyses were performed to identify the significant risk factors predicting depression and fear of COVID-19 scores. Among the general population sample, gender, marital status, and having chronic physical diseases were found to be the significant predictors (all *p* values <.001) for depression and fear of COVID-19. Having children was also a risk factor for depression, while depression was a risk factor for fear of COVID-19 (*p* < .001; Table 4). Among the general population, these factors explained 9% of the variance for depression and 26.6% of the variance for fear of COVID-19 [depression: *F* (4, 2549) = 64.324, *p* < .001; fear of COVID-19: *F* (5, 2548) = 184.811, *p* < .001].

**Table 4:** Multiple linear regression analysis identifying risk factors associated with depression and fear of COVID-19 among the general population sample (*n* = 2554)

Variables	Depression*				Fear of COVID-19**			
	β	95% CI		*p*-value	β	95% CI		*p*-value
		Lower bound	Upper bound			Lower bound	Upper bound	
*Constant*	–	3.923	5.230	<.001	–	11.598	13.452	<.001
Gender								
Female	.156	1.176	1.929	<.001	.117	.964	1.772	<.001
Male	Reference				Reference			
Marital status								
Married	−.115	−1.653	−.667	<.001	.092	.577	1.625	<.001
Unmarried	Reference				Reference			
Having children								
No	.090	.418	1.405	<.001	–	–	–	–
Yes	Reference							
Having chronic physical diseases								
Yes	.191	1.547	2.295	<.001	.073	.463	1.271	<.001
No	Reference				Reference			
Depression								
Yes	–	–	–	–	.455	.496	.578	<.001
No					Reference			

*R = 0.300, *R2* = 0.090, F (4, 2549) = 64.324 and *p* < .001

**R = 0.516, *R2* = 0.266, F (5, 2548) = 184.811 and *p* < .001

Among the HCPs, gender, being restless while examining a patient with flu-like symptoms, insecure feelings for oneself and family members due to COVID-19, and attitude toward a patient returning from abroad were found to be significant predictors (all *p*-values <.05) for depression and fear of COVID-19 (Table 5). Other risk factors for depression were marital status, needing to reuse PPE, and duration of using a single PPE (all *p*-values <.05) while for fear of COVID-19, the factor depression was the biggest contributor, *β* = .497 (all *p*-values <.001). Having no children and not being satisfied with the PPE quality negatively contributed to the fear of COVID-19 model. Among the HCP population, these factors explained 17.6% of the variance for depression and 45.6% of the variance for fear of COVID-19 [depression: *F*(9, 719) = 17.109, *p* < .001; fear of COVID-19: *F* (7, 721) = 86.251, *p* < .001]. An additional regression model also demonstrated that the fear of COVID-19 was a significant risk factor for depression status among participants (Table 6). More specifically, the factors in the models explained 27.7% of the variance among the general population and 39.6% of the variance among the HCP population.

**Table 5:** Multiple linear regression analysis identifying risk factors with depression and the fear of COVID-19 among the healthcare professional sample (*n* = 834)

Variables	Depression*				Fear of COVID-19**			
	β	95% CI		*p*-value	β	95% CI		*p*-value
		Lower bound	Upper bound			Lower bound	Upper bound	
*Constant*	–	.980	3.646	<.001	–	12.182	14.708	<.001
Gender								
Female	.196	1.265	2.589	<.001	.134	.879	2.139	<.001
Male	Reference				Reference			
Marital status								
Married	−.089	−1.623	−.234	.009	–	–	–	–
Unmarried	Reference							
Having children								
No	–	–	–	–	−.060	−1.282	−.063	.031
Yes					Reference			
Need to reuse PPE								
Yes	.098	.289	1.627	.005	–	–	–	–
No	Reference							
Duration of using single PPE								
A week	.104	.457	2.295	.003	–	–	–	–
More than a week	.091	.214	1.743	.012	–	–	–	–
Less than a week	Reference							
Restless when examining a patient with flu symptoms								
Yes	.148	.790	2.233	<.001	.215	1.848	3.211	<.001
No	Reference				Reference			
Satisfaction with PPE quality								
No	–	–	–	–	−.057	−1.644	−.017	.045
Yes					Reference			
Insecure feeling of oneself and family members due to COVID-19								
Yes	.102	.568	2.911	.004	.120	1.260	3.451	<.001
No	Reference				Reference			
Attitude toward a patient returning from abroad								
Felt restless	.121	.540	1.968	<.001	–	–	–	–
Maintain a distance of at least 1 m	–	–	–	–	−.078	−1.488	−.265	.005
No difference	Reference				Reference			
Having chronic physical diseases								
Yes	.150	.823	2.164	<.001	–	–	–	–
No	Reference							
Depression								
Yes	–	–	–	–	.497	.504	.637	<.001
No					Reference			

*R = 0.420, *R*^*2*^ = 0.176, *F*_*(9, 719)*_ = 17.109 and *p* < .001

**R = 0.675, *R*^*2*^ = 0.456, *F*_*(7, 721)*_ = 86.251 and *p* < .001

**Table 6:** Multiple linear regression models showing fear of COVID-19 as a significant risk factor for depression between in the study populations

Variables	General population (*n* = 716)*				Healthcare professional (*n* = 228)**			
	β	95% CI		*p*-value	β	95% CI		*p*-value
		Lower bound	Upper bound			Lower bound	Upper bound	
*Constant*	–	−2.379	−.782	<.001	–	−6.507	−4.024	<.001
Fear of COVID-19	.450	.352	.411	<.001	.558	.433	.538	<.001
Gender								
Female	.066	.312	1.00	<.001	.069	.092	1.269	.023
Male	Reference				Reference			
Marital status								
Married	−.134	−1.791	−.912	<.001	–	–	–	–
Unmarried	Reference							
Having children								
No	.101	.579	1.460	<.001	.089	.302	1.430	.003
Yes	Reference				Reference			
Having elderly people living at home who were at high-risk of COVID-19								
Yes	.046	.134	.825	<.001	.059	.010	1.294	.047
No	Reference				Reference			
Having chronic physical diseases								
Yes	.117	.832	1.509	<.001	.085	.270	1.436	.004
No	Reference							
Need to reuse PPE								
Yes	–	–	–	–	.089	.300	1.445	.003
No					Reference			
Duration of using single PPE								
A week	–	–	–	–	.083	.312	1.884	.006
More than a week	–	–	–	–	.058	−.025	1.283	.059
Less than a week					Reference			

*R = 0.526, *R*^*2*^ = 0.277, *F*_*(6, 2547)*_ = 162.537 and *p* < .001

**R = 0.629, *R*^*2*^ = 0.396, *F*_*(8, 720)*_ = 59.035 and *p* < .001

## Discussion

In Bangladesh, discussing mental health issues is still considered a taboo subject (The New Age 2019). Consequently, during the COVID-19 epidemic, individuals may suffer more because they feel they cannot open up and ask for help. Given that mental health issues among the general population and healthcare professionals (HCPs) have not been explored in Bangladesh (The Daily Star 2020), the present study addressed this knowledge gap and examined the potential associations between depression and fear due to COVID-19 among the two groups.

Approximately one-quarter of the participants were found to have depression (27.8%), and females were more prone to it among both in the general population and the HCPs. This finding is consistent with previous reports published outside of a pandemic situation (Moskvina et al. 2008; Silverstein 2002; Smith et al. 2008). In Bangladeshi culture, female HCPs tended to have work-family conflicts because they tend to be more responsible for their family, children, and patients. Additionally, they may experience more tension than males between career and family demands. This dilemma of trying to achieve the ideal work-life balance can make females feel they are failing which may increase their vulnerability to depression (Mone et al. 2019). In addition, and similar to a previous study during the SARS outbreak (Chan and Chan 2004), the findings in the present study showed that unmarried HCPs experienced more severe depression compared to those who were married.

The participants (among both the general population and HCPs) with depressive symptoms had significant fear of COVID-19. This may be due to the unpredictability, uncertainty, seriousness of the disease, fear of being infected, information gaps, and social isolation that have been created as a result of the epidemic (Furer et al. 1997; Zandifar and Badrfam 2020). Additionally, another study from Japan highlighted the role of economic factors influencing high levels of fear among patients, general population, and healthcare workers (Shigemura et al. 2020). In the present study, having one or more underlying medical conditions appeared to be a factor significantly contributing to depression and fear of COVID-19 among the population and is likely because they are more high risk of contracting the virus because of severe illness (Centers for Disease Control and Prevention 2020). Hopelessness, loneliness, anger, and the belief of pandemic not to be controlled may also be possible further causes of fear of death due to COVID-19. Previous studies have found that death anxiety plays a significant role in depression (Menzies et al. 2019). Furthermore, excessive fear of death due to COVID-19 has contributed to frustration, acute stress, self-harm, and suicide in some cases (Sahoo et al. 2020).

During the COVID-19 pandemic, many HCPs will have to face complex psychological issues due to the fear of getting infected with the deadly virus because there are limited resources such as personal protective equipment (PPE) (Ranney et al. 2020). Another study found that among 1257 healthcare workers working with COVID-19 patients in China*,* half of them (50.4%) had symptoms of depression (Lai et al. 2020). The present study found that the HCPs who had to reuse the same PPE for a week were in greater fear of COVID-19 than the HCPs who had to reuse the same PPE for less than a week. Likewise, in Bangladesh, approximately 25% of doctors and nurses and 60% of supporting staff involved in treating COVID-19 patients had not received PPE according to media reports (Al Jazeera 2020). Moreover, nurses are more discriminated during PPE distribution not only in Bangladesh (The Financial Express 2020a) but also in other parts of the world (Nursing Standard 2020; Think Global Health 2020). Additionally, the quality of PPE has been found to be questionable in some Bangladeshi healthcare facilities (The Financial Express 2020b; Ullah 2020) which based on the findings of the present study might lead to more mental health problems.

Also, the findings of the present study suggest that HCPs who felt restless while examining a patient with flu symptoms were more likely to be depressed and be in greater fear of COVID-19. This is unsurprising given that both flu and COVID-19 both have common symptoms such as fever, cough, cold, and runny nose (The Business Standard 2020). Additionally, not getting proper PPE may make the situation worse. In some cases, HCPs refused to provide treatment to the patients because of this (Hasan 2020) and may be a reflection of extreme fear. It has also been reported in the Bangladeshi media that some patients have concealed their medical symptoms and travel history to avoid stigma, social isolation, or quarantine which later forced HCPs to go into mandatory quarantine when these patients tested positive for COVID-19 fearing they had caught the virus (Al Jazeera 2020). HCPs who felt insecure about themselves and their family members had greater fear of COVID-19 compared to those that did not. This may be because they think that they might infect their family members (The New York Times 2020). This has terrified HCPs to take steps such as isolating themselves (Hartford Courant 2020; Mamun and Irani 2020) to reduce the risk of exposure.

The present study also highlights the gap between what can be afforded and provided at a given hospital and the actual needs of healthcare workers in Bangladesh during the COVID-19 pandemic. There is also a lack of COVID-19-related therapeutic interventions both for the general population and HCPs. Previous studies have highlighted the need of mental healthcare professionals in critical care units to minimize stress levels and reduce depression (Liu et al. 2020) while another study reported the positive impact of telephone helplines for HCPs to address mental health problems (Kang et al. 2020). Most HCPs in the present study similarly believed that they were unprepared to tackle the current pandemic and said that psychological interventions were important in the current pandemic.

A paper from India discussed the importance of assigning a psychiatrist during the COVID-19 pandemic and included strategies including educating the public about the effects of a pandemic, motivating them to implement disease prevention policies, integrating available healthcare-related services with the existing settings, empowering patients with COVID-19, and providing psychological interventions to HCPs (Banerjee 2020). The general public can also follow the strategies suggested by a recently published paper from a Bangladesh perspective to tackle fear due to COVID-19 (Mamun and Griffiths 2020) such as avoiding untrustworthy news and information sources, offering support and signposting for individuals who may be experiencing mental health problems, and providing help in the lockdown to those most in need. In response to the pandemic, the Bangladeshi government initially implemented a number of initiatives such as closing educational institutions (and introduced online teaching), prohibiting any type of social, political or religious marches, limiting banking services, closing all non-essential private and public offices as part of the national lockdown, and restricting entry to the country. However, the country has more challenges to tackle. For instance, the testing rate for COVID-19 is still one of the lowest among the South Asian nations (Chowdhury et al. 2020). Moreover, there is a lack of intensive care unit beds and oxygen cylinders in the hospitals which is of great concern. Therefore, the country needs the necessary healthcare facilities and support including intensive care unit beds, ventilators, high-grade personal protective equipment, sufficient testing kits, proper training for HCPs who are assigned in COVID-19 dedicated hospital, and increased funds to overcome the COVID-19 pandemic.

## Limitations

The present study has a number of limitations due to the cross-sectional nature as well as other methodological biases including convenience sampling and non-representative self-reported data. Owing to the lockdown situation in Bangladesh, it was not possible to collect the data in person. Because of this, an online platform was used to recruit participants to complete the survey. Consequently, only individuals who were able to use the internet were likely to respond. Therefore, the study did not include many individuals of low socioeconomic status because the poor are unable to afford internet access. Their opinions and levels of depression were therefore not addressed in the study. Moreover, individuals who had pre-existing depression were not considered separately when predicting the fear of COVID-19 in the regression analysis. It is possible that there is a bi-directional relationship between fear of COVID-19 and depression.

## Conclusions

One of the major causes of stress and depression among individuals at the current time is the unpredictability of the COVID-19 situation and when the disease will be under control. It is apparent in the present study that the depression levels of the frontline healthcare professionals are similar to the general public but there may be different underlying proximal reasons for the depression. Among the general population, levels of depression may get worse due to the lack of social interaction and feeling isolated in their own homes during quarantine. For HCPs, the working situation they find themselves in may be a much bigger contributor to poor mental health. Measures to promote mental wellbeing in both the general population and medical workers need to be promptly executed, with women, the elderly, and frontline workers demanding particular attention.
